# Combining CHAMP and Swarm Satellite Data to Invert the Lithospheric Magnetic Field in the Tibetan Plateau

**DOI:** 10.3390/s17020238

**Published:** 2017-01-26

**Authors:** Yaodong Qiu, Zhengtao Wang, Weiping Jiang, Bingbing Zhang, Fupeng Li, Fei Guo

**Affiliations:** 1 School of Geodesy and Geomatics, Wuhan University, 129 Luoyu Road, Wuhan 430079, China; ydqiu@whu.edu.cn (Y.Q.); ztwang@sgg.whu.edu.cn (Z.W.); bbzhang@whu.edu.cn (B.Z.); fpli@whu.edu.cn (F.L.); fguo@whu.edu.cn (F.G.); 2 GNSS Research Center of Wuhan University, 129 Luoyu Road, Wuhan 430079, China

**Keywords:** Lithospheric Magnetic Field, geological structures, Swarm satellite, CHAMP satellite, R-SCHA

## Abstract

CHAMP and Swarm satellite magnetic data are combined to establish the lithospheric magnetic field over the Tibetan Plateau at satellite altitude by using zonal revised spherical cap harmonic analysis (R-SCHA). These data are integrated with geological structures data to analyze the relationship between magnetic anomaly signals and large-scale geological tectonic over the Tibetan Plateau and to explore the active tectonic region based on the angle of the magnetic anomaly. Results show that the model fitting error is small for a layer 250–500 km high, and the RMSE of the horizontal and radial geomagnetic components is better than 0.3 nT. The proposed model can accurately describe medium- to long-scale lithospheric magnetic anomalies. Analysis indicates that a negative magnetic anomaly in the Tibetan Plateau significantly differs with a positive magnetic anomaly in the surrounding area, and the boundary of the positive and negative regions is generally consistent with the geological tectonic boundary in the plateau region. Significant differences exist between the basement structures of the hinterland of the plateau and the surrounding area. The magnetic anomaly in the Central and Western Tibetan Plateau shows an east–west trend, which is identical to the direction of the geological structures. The magnetic anomaly in the eastern part is arc-shaped and extends along the northeast direction. Its direction is significantly different from the trend of the geological structures. The strongest negative anomaly is located in the Himalaya block, with a central strength of up to −9 nT at a height of 300 km. The presence of a strong negative anomaly implies that the Curie isotherm in this area is relatively shallow and deep geological tectonic activity may exist.

## 1. Introduction

The lithospheric magnetic field accounts for approximately 4% of the total energy of the earth’s magnetic field [1]. It originates from the crust and upper mantle of magnetic rocks and reflects a 3D spatial distribution of rock magnetism. Given its different rock magnetic environments, temperature pressure conditions, magnetic carriers, and tectonic evolutions, the lithospheric magnetic field carries abundant and complex geological structures and tectonic movement information [2,3]. Therefore, analyzing the magnetic distribution frame in the depth of the crust and the top of the upper mantle using a high-resolution and high-accuracy lithospheric magnetic field model can reveal important information regarding regional tectonics and their evolution, the division of rock masses, and the heat flow of the lithosphere [4,5,6,7,8].

The accuracy of lithospheric magnetic field models and spatial resolutions improved significantly with the recent accumulation of Øersted, CHAMP, Swarm, and other satellite magnetic data, as well as the continuous improvement of inversion algorithms and separation technology of the various source fields [9]. A series of representative lithospheric magnetic field models was successively released internationally. The minimum spatial wavelength reaches 130 km when satellite observations are only used for the recently released HDGM-2016 model (http://www.ngdc.noaa.gov/geomag/HDGM/index.html). The minimum spatial wavelength is approximately 300 km when the spherical harmonic function expansion is 133 degrees for the POMME-10 model (http://geomag.org/models/pomme10.html). Unlike ground and aero magnetic surveys, satellite magnetic survey is characterized by high coverage and repetition rate; this approach can be used to observe the change of the earth’s magnetic field in real time [10,11]. The altitude of satellite observation data is relatively high. Thus, observation data can effectively suppress the interference of surface environments and shallow geological bodies. Furthermore, the medium/long-wavelength signal of the lithospheric magnetic field was extracted and used in studies on tectonic structures in the deep crust and the top of the upper mantle, as well as the depth of the Curie surface and heat flow anomaly [12,13,14,15]. Vervelidou et al. calculated the magnetization intensity and the thickness of a global large-scale lithospheric magnetic field using a revised spherical cap harmonic model to analyze the spatial power spectrum of the lithosphere magnetic field [16]. Bouligand et al. estimated the depth of magnetic sources by analyzing the radial power spectrum of aeromagnetic anomaly by assuming that magnetization has a fractal distribution; a fractal crustal magnetization model was used to map the depth of the Curie. An obvious corresponding relationship between the characteristics of the bottom of magnetic sources and the prominent heat flow anomalies was identified. The study provided new information regarding geological structures and heat flow anomalies [17]. Ou et al. (2013) found that the Z component of the satellite magnetic anomalies was consistent with the geological features of the old craton in China. Moreover, the relationship between the geological detail features and the radial component of magnetic anomalies was thoroughly explained by integrating ground magnetic survey data [18]. Maule used satellite magnetic data to estimate the heat flow underneath the Antarctic ice sheet. With the enhancement of the resolution of the satellite magnetic field models, the method is believed to be capable of identifying the detailed features of heat flow and may be utilized to eventually discover volcanoes that have not yet been discovered beneath the glacier [12].

The spatial resolution of a magnetic anomaly model based on satellite magnetic data is mainly influenced by two factors. First, small-scale magnetic anomaly signals, which decay with the altitude for spaceborne sensor devices, are difficult to capture [19]. Second, the sensors are strongly influenced by the external magnetic field at satellite altitude, and the magnitude even exceeds the intensity of the magnetic field signal itself. Therefore, the interference of the external field should be excluded from the modeling process of magnetic anomalies. Two common modeling methods are available [20], as follows: comprehensive inversion (CI) and sequential modeling. CI utilizes comprehensive mathematical models to describe the sources only in time and space on the average and on the inversion of all types of field sources using iteratively reweighted least-squares [21,22,23]. However, the time-varying characteristic of the external field is evident in the repeat period of a satellite orbit. The time-varying signal may overlap with the weak small-scale crustal magnetic anomaly signal. Thus, the time-varying signal may be difficult to distinguish. The sequential approach selects the observed data with less external field interference and revises the selected data using an established empirical model. Furthermore, along-track filter processing is conducted for the revised data. Finally, inversion modeling is conducted for the refined data [24]. However, this method may induce spectral leakage in the process of correcting the external field.

Regional modeling exhibits better fitting effect on the fine structures of geomagnetic anomalies than spherical harmonic analysis when the spatial distribution of regional magnetic anomalies is studied [8]. The advantages of regional modeling are evident when satellite observation data are densely distributed across layers of different heights or are unevenly distributed in a horizontal direction. Common regional modeling methods mainly include the equivalent source method [25], rectangular harmonic analysis [26,27], the spherical cap harmonic/revised spherical cap harmonic analysis (R-SCHA) method [28,29,30,31], and the 3D Taylor/Legendre polynomial method [32,33,34]. These methods have various advantages and disadvantages. Using CHAMP and Swarm satellite magnetic data, the current study utilizes Sq local time conditions, Dst/Kp geomagnetic index conditions, Hamming along-track high-pass filtering, and the hierarchical gridding method, and combines EMM2015, MF7 and other auxiliary models to thoroughly process satellite magnetic data. The present paper adopts zonal revised spherical cap harmonic analysis to inverse the lithospheric magnetic field over the Tibetan Plateau and analyzes the relationship between magnetic anomalies and geological structures to explore the active region of geological structures.

## 2. Mathematical Model and Inversion Method

### 2.1. Mathematical Model

**Figure 1:**
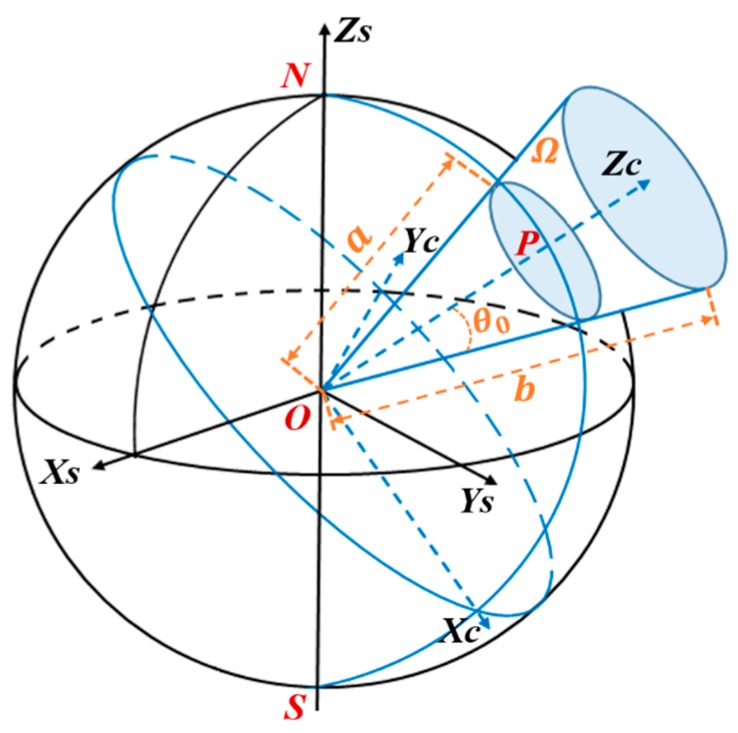
Sketch of the spherical cap coordinate system: Magnetic field represented in a conical domain Ω bounded by a lower and an upper spherical surfaces with radii a and b, respectively. $\theta_{0}$ is half aperture of the cone. P is pole of the cone. ${\left(X,Y,Z\right)}_{S}$ are geocentric spatial rectangular coordinate system based on sphere. ${\left(X,Y,Z\right)}_{C}$ are geocentric spatial rectangular coordinate system based on spherical cap. N,S,O are the north pole, the south pole, and the center of the earth, respectively.

The model is established in a source-free conical domain denoted by Ω, which is bounded by a finite cone semi-angle of $\theta_{0}$. The domain lower and upper boundaries along the radial r are defined with two spherical surfaces of radii a and b, respectively, as shown in Figure 1. The boundary of cone Ω was defined as follows: $\partial \Omega =\;\partial_{\theta_{0}}\Omega \cup \partial_{a}\Omega \cup \partial_{b}\Omega$. $\partial_{\theta_{0}}\Omega :\left\{\theta =\theta_{0},\;a\leq r\leq b\right\}$, $\partial_{a}\Omega :\left\{\theta \leq \theta_{0},\;r=a\right\}$ and $\partial_{b}\Omega :\left\{\theta \leq \theta_{0},\;r=b\right\}$ represent the lateral boundary, the lower and upper boundary of Ω, respectively [18,30]. Suppose that the geomagnetic field meets the irrotational and solenoidal requirements in the closed region Ω. The geomagnetic field scalar potential meets the Laplace equation $\nabla^{2}V=0$ under the condition of boundary constraint. By using the separation of variables method, the solution of Laplace’s equation can be written as a sum of infinite series in a cone coordinate system [30]:

(1) $$\begin{array}{ll} V(r,\theta ,\varphi )= & a\sum_{k\geq 0}\sum_{m\geq 0}{\left(\frac{a}{r}\right)}^{n_{k}+1}\left(G_{n_{k}}^{i,m}\cos(m\varphi )+H_{n_{k}}^{i,m}\sin(m\varphi )\right)\cdot P_{n_{k}}^{m}(\cos\theta )\\ & +\;a\sum_{k\geq 0}\sum_{m\geq 0}{\left(\frac{r}{a}\right)}^{n_{k}}\left(G_{n_{k}}^{e,m}\cos(m\varphi )+H_{n_{k}}^{e,m}\sin(m\varphi )\right)\cdot P_{n_{k}}^{m}(\cos\theta )\\ & +\;a\sum_{p\geq 1}\sum_{m\geq 0}R_{p}(r)\left(G_{p}^{m}\cos(m\varphi )+H_{p}^{m}\sin(m\varphi )\right)\cdot K_{p}^{m}(\cos\theta )\\ & +\;a\sum_{m\geq 0}R_{0}\left(G_{0}^{m}\cos(m\varphi )+H_{0}^{m}\sin(m\varphi )\right)\cdot P_{0}^{m}(\theta ) \end{array}$$

The corresponding expressions for the geomagnetic component are as follows [18,31]:

(2) $$\begin{array}{l} \begin{array}{ll} X= & {\sum_{k\geq 0}\sum_{m\geq 0}\left(\frac{a}{r}\right)}^{n_{k}+2}\frac{dP_{n_{k}}^{m}}{d\theta }\left(G_{n_{k}}^{i,m}\cos\left(m\varphi \right)+H_{n_{k}}^{i,m}\sin\left(m\varphi \right)\right)\\ & +{\sum_{k\geq 0}\sum_{m\geq 0}\left(\frac{r}{a}\right)}^{n_{k}-1}\frac{dP_{n_{k}}^{m}}{d\theta }\left(G_{n_{k}}^{e,m}\cos\left(m\varphi \right)+H_{n_{k}}^{e,m}\sin\left(m\varphi \right)\right)\\ & +\frac{a}{r}\sum_{p\geq 1}\sum_{m\geq 0}R_{p}\left(r\right)\frac{dK_{p}^{m}}{d\theta }\left(G_{p}^{m}\cos\left(m\varphi \right)+H_{p}^{m}\sin\left(m\varphi \right)\right)\\ & +\frac{a}{r}\sum_{m\geq 0}R_{0}\frac{dP_{0}^{m}}{d\theta }\left(G_{0}^{m}\cos\left(m\varphi \right)+H_{0}^{m}\sin\left(m\varphi \right)\right) \end{array}\\ \begin{array}{ll} Y= & {\sum_{k\geq 0}\sum_{m\geq 1}\left(\frac{a}{r}\right)}^{n_{k}+2}\frac{mP_{n_{k}}^{m}}{\sin\theta }\left(G_{n_{k}}^{i,m}\sin\left(m\varphi \right)-H_{n_{k}}^{i,m}\cos\left(m\varphi \right)\right)\\ & +{\sum_{k\geq 0}\sum_{m\geq 1}\left(\frac{r}{a}\right)}^{n_{k}-1}\frac{mP_{n_{k}}^{m}}{\sin\theta }\left(G_{n_{k}}^{e,m}\sin\left(m\varphi \right)-H_{n_{k}}^{e,m}\cos\left(m\varphi \right)\right)\\ & +\frac{a}{r}\sum_{p\geq 1}\sum_{m\geq 1}R_{p}\left(r\right)\frac{mK_{p}^{m}}{\sin\theta }\left(G_{p}^{m}\sin\left(m\varphi \right)-H_{p}^{m}\cos\left(m\varphi \right)\right)\\ & +\frac{a}{r}\sum_{m\geq 1}R_{0}\frac{mP_{0}^{m}}{\sin\theta }\left(G_{0}^{m}\sin\left(m\varphi \right)-H_{0}^{m}\cos\left(m\varphi \right)\right) \end{array}\\ \begin{array}{ll} Z= & -{\sum_{k\geq 0}\sum_{m\geq 0}\left(n_{k}+1\right)\left(\frac{a}{r}\right)}^{n_{k}+2}P_{n_{k}}^{m}\left(G_{n_{k}}^{i,m}\cos\left(m\varphi \right)+H_{n_{k}}^{i,m}\sin\left(m\varphi \right)\right)\\ & +{\sum_{k\geq 0}\sum_{m\geq 0}n_{k}\left(\frac{r}{a}\right)}^{n_{k}-1}P_{n_{k}}^{m}\left(G_{n_{k}}^{e,m}\cos\left(m\varphi \right)+H_{n_{k}}^{e,m}\sin\left(m\varphi \right)\right)\\ & +\sum_{p\geq 1}\sum_{m\geq 0}\frac{dR_{p}\left(r\right)}{dr}K_{p}^{m}\left(G_{p}^{m}\cos\left(m\varphi \right)+H_{p}^{m}\sin\left(m\varphi \right)\right) \end{array} \end{array}$$

In Equation (2), X,Y,Z represent the north, east, and vertical downward components of the geomagnetic field at an observation station, respectively. a denotes the mean radius of the earth, which is taken to be 6371.2 km. φ,θ,r represent the longitude, geocentric colatitude, and geocentric distance, respectively, in the cone coordinate system. $P_{n_{k}}^{m}(\cos\theta )$ is the associated Schmidt quasi-normalization Legendre functions of real degree $n_{k}$ and integer order m. $K_{p}^{m}(\cos\theta )$ is the Mehler functions. $P_{0}^{m}(\cos\theta )$ is the particular form of the $K_{p}^{m}(\cos\theta )$ function when n≤m and n=0. $R_{p}(r)$ are the radial functions. $R_{0}=\sqrt{a}/\sqrt{b-a}$ is the particular form of the $R_{p}(r)$ function when n=0 [35]. $\left(G_{n_{k}}^{i,m},H_{n_{k}}^{i,m},G_{n_{k}}^{e,m},H_{n_{k}}^{e,m}\right)$ are the Legendre coefficients, and $\left(G_{p}^{m},H_{p}^{m},G_{0}^{m},H_{0}^{m}\right)$ are the Mehler coefficients. k,p are integers that correspond to the sequence number of roots $n_{k},n_{p}$ Superscripts i,e of the Legendre coefficient refer to the components of the internal and external source field. Achieving the separation of the internal and external field sources is more difficult when the R-SCHA algorithm is used with the aid of the boundary conditions of cone Ω compared with spherical harmonic analysis. Therefore, careful selection and model correction are conducted on the satellite data to exclude the interference of external source fields as much as possible. The component of the external source field introduced in the algorithm represents neither the ionosphere nor the magnetic field. Instead, it adopts two groups of Legendre coefficients to fit the distribution form of the geomagnetic field in cone Ω. According to radial function form (r/a) corresponding to the Legendre coefficient $\left(G_{n_{k}}^{e,m},H_{n_{k}}^{e,m}\right)$ in the formula (2), we can infer that $\left(G_{n_{k}}^{e,m},H_{n_{k}}^{e,m}\right)$ mainly describes the contribution of the geomagnetic field in the middle and upper space altitude layers and several long wavelength components of the geomagnetic field in the earth’s surface space. After testing, Torta et al. found that achieving the best fit is difficult using the model if the component of the so-called external source field was removed from the algorithm, but the independent component of the internal source field was only used even if the observation data was distributed in the lower bottom boundary of the spherical cap. The fitting effect improves significantly if the component of the external source field is added to the Legendre function [36,37].

### 2.2. Inversion Method

The observation vector of the field $L=\left\{l_{1},l_{2},\cdots l_{n}\right\}$ and parameter vector to be estimated (revised spherical cap harmonic coefficient) $X=\left\{x_{1},x_{2},\cdots ,x_{k}\right\},\;n>>k$, satisfy the following linear relation:

(3) L=AX+Δ.

In Equation (3), Δ represents the observation noise. Its stochastic characteristic is $E(\Delta )=0,\;D(\Delta )=\sigma_{0}^{2}{P_{L}}^{-1}$ where $P_{L}$ represents the observation vector weight matrix and $\sigma_{0}$ denotes the mean square error of unit weight.

A target function is constructed according to the estimation criterion of the least square principle of compensation to provide a unique stable resolution for Equation (3):

(4) $$J_{\alpha }(L,X)={\left\|AX-L\right\|}_{P_{L}}^{2}+\alpha \cdot \Omega (X).$$

Parameter vector $\hat{X}$ that meets the minimization of Equation (4) is the solution of the linear model by Equation (3). Ω(X) is called a stable function. It functions to transfer ill-posed problems to well-posed problems. α represents the regularization parameter (smoothing factor). It functions to balance the weights of two items of target function $J_{\alpha }(L,X)$. ${\left\|\;\cdot \;\right\|}_{P_{L}}^{2}$ represents the weighted Euclidean two-norm [38]. In practice, stable functional Ω(X) typically uses the Tikhonov regularization method with the following form [39,40,41,42]:

(5) $$\Omega (X)={\left\|X\right\|}_{P_{X}}^{2}=X^{T}P_{X}X.$$

In Equation (5), $P_{X}$ represents the parameter weight matrix. For the above stable functional, the minimum value of target function $J_{\alpha }(L,X)$ is calculated as follows:

(6) $$J_{\alpha }(L,X)={\left\|AX-L\right\|}_{P_{L}}^{2}+\alpha \cdot X^{T}P_{X}X=\min.$$

If $\partial J_{\alpha }(L,X)/\partial X=0$, then a parametric solution can be obtained as follows:

(7) $$X={\left(A^{T}P_{L}A+\alpha P_{X}\right)}^{-1}A^{T}P_{L}L.$$

Based on the abovementioned analysis, the selection of reasonable parameter weight function $P_{X}$ is the key to solving ill-posed problems. Given that the Legendre and Mehler functions are mutually orthogonal, parameter weight function $P_{X}$ can be set as a diagonal matrix. The elements in the diagonal line of matrix $P_{X}$ can be determined by geomagnetic energy per coefficient. Besides the additional constraint or a stable functional Ω(X), regularization parameter α also plays an important role in ill-posed problems [43]. α balances the influence of data fitting degree and regularized error for parameter estimation [43]. The optimal regularization parameter should minimize the comprehensive error caused by both factors. When α is excessively small, the inversion model tends to fit the observation data effectively and with a small constraint effect, which may induce over-fitting of high-frequent noise in the observation data. As a result, the solution of target function loses physical meaning because of shock. If α is excessively large, then the inversion model will be inclined to constraints, thereby leading to excessive smoothing of the model parameters. As a result, high-frequency information is lost, and the fitting effect of observation data is poor.

To estimate the optimal regularization parameter a, Thébault et al. used CM4 synthetic data to calculate the correlation coefficient between the R-SCHA prediction and the expected magnetic field and in the data gap layer at a height of 100~300 km. After testing, set $\alpha =1e^{-6}$ was identified for the internal Legender functions. Set $\alpha =9e^{-7}$ was determined for the Mehler functions. The R-SCHA algorithm can provide the best balance between the fit and the smoothness of the edges, as well as the reliability of the data gap layer [31]. By using multiple simulation data, Thébault and Gaya–Piqué (2008) found that the model can also provide a stable space continuation in equal weight without considering regularization conditions. From a side view, when the truncation level of a model is reasonably designed, the R-SCHA algorithm is stable for local regional modeling without obvious shock effects [44].

### 2.3. Coordinate Transformation

Before model inversion, the geographical coordinates ${\left(\varphi ,\theta ,r\right)}_{S}$ and geomagnetic component ${\left(N,E,U\right)}_{S}$ of the observation data must be transformed from the spherical coordinate reference frame to the cone coordinate reference frame [45], as shown in Figure 1.

#### 2.3.1. Transformation of Geographical Coordinates

The geographical coordinate of the observation data ${\left(\varphi ,\theta ,r\right)}_{S}$ is transformed from the spherical coordinate system to the cone coordinate system. The transformation is conducted in three steps:

(1) In the spherical coordinate reference frame, Equation (8) is used to transform the geographical coordinate ${\left(\varphi ,\theta ,r\right)}_{S}$ to the earth’s core space rectangular coordinate system ${\left(X,Y,Z\right)}_{S}$;

(8) $${\left[\begin{array}{c} X\\ Y\\ Z \end{array}\right]}_{S}={\left[\begin{array}{c} \cos\theta \cdot \cos\varphi \\ \cos\theta \cdot \sin\varphi \\ \sin\theta \end{array}\right]}_{S}.$$

(2) Equation (9) is utilized to transform ${\left(X,Y,Z\right)}_{S}$ to the cone space rectangular coordinate system ${\left(X,Y,Z\right)}_{C}$;

(9) $${\left[\begin{array}{c} X\\ Y\\ Z \end{array}\right]}_{C}=R_{y}(\theta_{0})\cdot R_{z}(\varphi_{0})\cdot {\left[\begin{array}{c} X\\ Y\\ Z \end{array}\right]}_{S}.$$

In Equation (9), $R_{y}(\theta_{0})=\left[\begin{array}{ccc} \cos\theta_{0} & 0 & -\sin\theta_{0}\\ 0 & 1 & 0\\ \sin\theta_{0} & 0 & \cos\theta_{0} \end{array}\right]\;R_{z}(\varphi_{0})=\left[\begin{array}{ccc} \cos\varphi_{0} & \sin\varphi_{0} & 0\\ -\sin\varphi_{0} & \cos\varphi_{0} & 0\\ 0 & 0 & 1 \end{array}\right]$, and $\left(\varphi_{0},\theta_{0}\right)$ refers to the longitude and geocentric colatitudes of the cone pole;

(3) In the cone coordinate reference frame, Equation (10) is used to transform ${\left(X,Y,Z\right)}_{C}$ to the cone geographical coordinate system ${\left(\varphi ,\theta ,r\right)}_{C}$:

(10) $${\left[\begin{array}{c} \varphi \\ \theta \end{array}\right]}_{C}={\left[\begin{array}{c} \arctan\frac{Y}{X}\\ \arcsin\frac{Z}{r} \end{array}\right]}_{C}.$$

#### 2.3.2. Transformation of Geomagnetic Observation Component

The transformation of geomagnetic observation component ${\left(N,E,U\right)}_{S}$ is conducted through three steps:

(1) In the spherical coordinate reference frame, Equation (11) is used to transform the geomagnetic observation component ${\left(N,E,U\right)}_{S}$ in station center coordinate system to the geocentric space rectangular coordinate system ${\left(X,Y,Z\right)}_{S-VAL}$;

(11) $${\left[\begin{array}{c} X\\ Y\\ Z \end{array}\right]}_{S-VAL}=\;\left[\begin{array}{ccc} -\sin B_{0}\cos L_{0} & -\sin L_{0} & -\cos B_{0}\cos L_{0}\\ -\sin B_{0}\sin L_{0} & \cos L_{0} & -\cos B_{0}\sin L_{0}\\ \cos B_{0} & 0 & -\sin B_{0} \end{array}\right]\cdot {\left[\begin{array}{c} N\\ E\\ U \end{array}\right]}_{S}$$

In Equation (11), $\left(B_{0},L_{0}\right)$ represent the geocentric latitude and longitude in the spherical coordinate system, respectively.

(2) Similarly, Equation (12) is used to transform ${\left(X,Y,Z\right)}_{S-VAL}$ into the cone space rectangular coordinate system ${\left(X,Y,Z\right)}_{C-VAL}$, namely:

(12) $${\left[\begin{array}{c} X\\ Y\\ Z \end{array}\right]}_{C-VAL}=R_{y}(\varphi )\cdot R_{z}(\lambda )\cdot {\left[\begin{array}{c} X\\ Y\\ Z \end{array}\right]}_{S-VAL}.$$

(3) In the cone coordinate reference frame, Equation (13) is used to transform ${\left(X,Y,Z\right)}_{C-VAL}$ to geomagnetic observation component ${\left(N,E,U\right)}_{C}$ in station center coordinate system.

(13) $${\left[\begin{array}{c} N\\ E\\ U \end{array}\right]}_{C}=\left[\begin{array}{ccc} -\sin B_{0}\cos L_{0} & -\sin B_{0}\sin L_{0} & \cos B_{0}\\ -\sin L_{0} & \cos L_{0} & 0\\ -\cos B_{0}\cos L_{0} & -\cos B_{0}\sin L_{0} & -\sin B_{0} \end{array}\right]\cdot {\left[\begin{array}{c} X\\ Y\\ Z \end{array}\right]}_{C-VAL}$$

(Note: in Equation (13), ${\left(N,E,U\right)}_{C}$ correspond to the geomagnetic observation component (X,Y,Z) in the spherical cap harmonic model in Equation (2)).

## 3. Data Source and Data Preprocessing

### 3.1. Data Source

**Figure 2:**
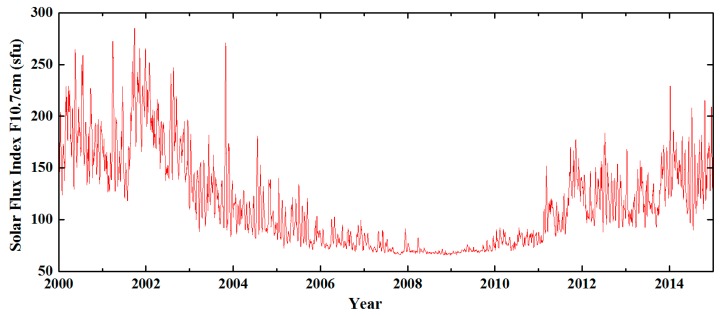
Solar radiation index F10.7 from 2000~2015.

The satellite data used in the present paper include: (1) CHAMP vector observation data from 2008.1~2010.9. The data in this period correspond to low solar activity years with a corresponding satellite orbital altitude of 250~340 km, as shown in Figure 2. Therefore, the data are less affected by the external magnetic field. This condition is conducive to the exploration of middle and small-scale magnetic anomaly signals. (2) Given that the Swarm C satellite orbit is excessively high to be sensitive to middle and small-scale magnetic anomaly signals, the vector observation data of Swarm A and B satellites from 2014.1~2015.12 with an orbital altitude of 450~510 km are selected. Satellite circular half-orbit separation and data are subsampled every 5s before data processing. Track separation is conducted along-track filtering corrections of observations. At satellite orbit altitude, sampling with 5 s intervals corresponds to a spacing of approximately 35 km. The sampling can weaken the reciprocal mixing of small-scale magnetic field signals and effectively control the anisotropic error to be introduced in the final model.

### 3.2. Data Screening

**Figure 3:**
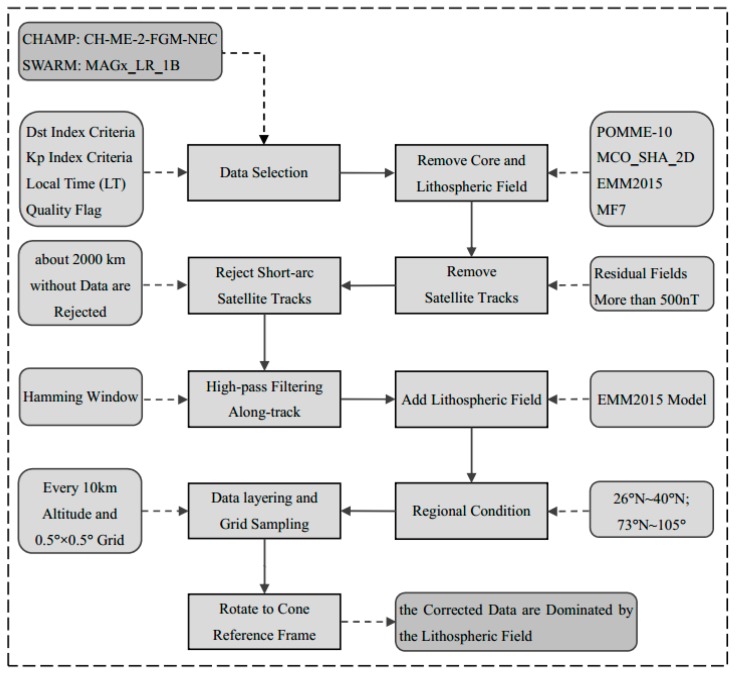
Flow chart showing the sequence of processing steps to separate the lithospheric field contributions from the satellite magnetic data.

To decrease the interference of the external source field in establishing lithospheric magnetic field model, according to the satellite data screening criteria proposed by Maus and Sabaka et al. [24,46], the data are selected in local time 22:00~03:00 to minimize the contributions from the ionospheric Sq field. The Dst and Kp index conditions in Equation (14) are utilized to select the data corresponding to a quiet period of geomagnetic activity to weaken the effects of the equatorial ring current and magnetospheric convection intensity:

(14) $$\left\{ \begin{array}{lll} \left|Dst\right|\leq 20nT & and & d\left|Dst\right|\leq 10nT\\ Kp\leq 2 & and & d|Kp|\leq 2 \end{array}\right.$$

d|Dst| and d|Kp| represent changes over the previous three hours. A selection condition is selected using quality flags indicators, such as spacecraft maneuvers and the on/off status of the star camera, to judge the running state of a star camera. Finally, short-arc tracks without data beyond 240 epochs are eliminated to fully guarantee the quality of the observed data. Figure 3 shows the data preprocessing procedure of extracting a lithospheric magnetic field signal based on satellite magnetic survey data.

### 3.3. Main Magnetic Field and External Magnetic Field Correction

**Figure 4:**
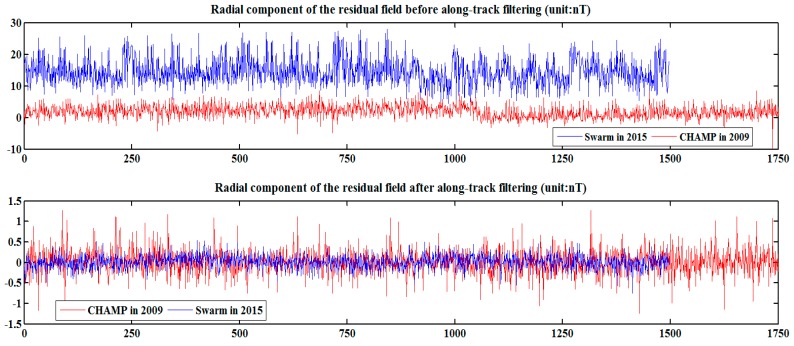
Z component of the residual field before and after correction of the CHAMP (in 2009) and Swarm (in 2015) satellite data. We used the MF7 model to remove the lithospheric field for this illustration.

The non-lithospheric magnetic field must be corrected for the selected data to capture lithospheric magnetic field signal [2]. The corrected magnetic fields cover the main magnetic field, magnetosphere, and ionospheric magnetic field, as well their induced counterparts. Considering the weakness of the ionospheric magnetic field at night, corrections are not conducted using the model. To correct the magnetospheric sources, the long wavelength signals are separated by means of along-track filtering in Section 3.4. To correct the main magnetic field, the POMME-10, EMM2015, and MCO_SHA_2D models were tested [47]. Their mutual differences are small. Therefore, the EMM2015 model (1–15 degrees) is selected as the earth’s main magnetic field and its secular variation. The model is removed from the satellite observation value to obtain the so-called residual field. Figure 4 describes the radial components of CHAMP and Swarm satellite observations after deleting the main magnetic field. The MF7 lithospheric magnetic field model is eliminated to comprehensively analyze the along-track filtering data in Section 3.4. Figure 4 shows that before making along-track filtering corrections, the residual magnetic field of CHAMP and Swarm observations range from −10~10 nT and 0~30 nT, respectively.

### 3.4. Along-Track Filtering

After data screening and main magnetic field correction, several non-lithospheric signals that were not modeled remain in the observations. These residual signals are dominated by long-wave components of magnetospheric contributions [20,48]. Another important source of residuals is the ionospheric disturbance magnetic field, and the sources of secondary importance are the induced magnetic field and the secular variation error from the main magnetic field. Along-track high-pass filtering of fast Fourier transform is adopted in the current study to eliminate the large-scale residual signals. The challenge in employing this method is the selection of the cut-off wavelength. The magnetic field contributions from wavelength signals greater than 1200 km are reduced with high pass filtering by using a Hamming window function (filter of order 128). In the study, a lithospheric field (MF7) is removed before performing along-track filtering for the residual signals in the actual filtering process to lower the level of spectral leakage [49]. Finally, a residual analysis is conducted for the values after filtering, and magnetic anomaly values greater than ±25 nT are eliminated. At satellite altitude, the intensity of lithospheric magnetic anomaly is generally less than 25 nT [50].

### 3.5. Data Hierarchical Gridding

Hierarchical gridding of the data is applied to avoid the shock effect of the model in the horizontal and vertical directions caused by the uneven data distributions. In the horizontal direction, the grid resolution of data is set to 0.5° × 0.5°. In the vertical direction, the layered interval of data is set to 10 km. A layer of insufficient data is merged into an adjacent layer. For the data distributed in the same grid unit, the data nearest the average value of the grid is selected as the magnetic anomaly value of the grid to ensure the equilibrium of the data in the horizontal and vertical directions.

## 4. Lithospheric Magnetic Field Model of the Tibetan Plateau

### 4.1. Related Parameter Setting

To achieve the best fit between the selected spherical cap and the modeling region, the pole of the spherical cap is generally set at the center of the region or nearby. The geographic latitude and longitude of the Tibetan Plateau are $26.0^{\circ }N\sim 40.0^{\circ }N,\;73.0^{\circ }E\sim 105.0^{\circ }E$, respectively. The difference of longitude is approximately twice the difference of latitude. Under a certain level of truncation, partition modeling of a small spherical cap can be used to improve the accuracy of the model in the whole region and generate good spatial resolution [51]. Therefore, the Tibetan Plateau was divided into two subregions, and each subregion was independently modeled. The pole of the spherical cap in Region 1 is located at $\left(33^{\circ }N,\;81^{\circ }E\right)$, and the pole of spherical cap in Region 2 is located at $\left(33^{\circ }N,\;97^{\circ }E\right)$. To suppress the boundary effect, the half aperture of the spherical cap was amplified of 1°, and $\theta_{1}=\theta_{2}{=10}^{\circ }$ was set. The largest overlap between adjacent spherical caps is approximately 7.8°. Thus, all the observed data are located within the range of the spherical cap.

The mean altitude of the Tibetan Plateau is greater than 4 km. The height of the lowest orbit of the selected CHAMP satellite data is approximately 250 km. The height of the highest orbit of Swarm satellite data is approximately 510 km. Therefore, the geocentric distances of the lower and upper boundaries of the cone are defined as a=Re+ 240km and b=Re+520km, where Re=6371.2km refers to the earth’s mean radius. The related parameters are listed in Table 1.

In Table 1, $K_{\max}^{i},K_{\max}^{e}$ represent the maximum truncation degree of the Legendre function in the internal and external source fields, respectively. $P_{\max}$ denotes the maximum truncation degree of the Mehler function. Comprehensive consideration of the data size in each layer and the magnetic sensors do not easily capture the weak middle- and small-scale lithospheric magnetic field signals with lengths less than 200 km at the CHAMP orbital altitude. Therefore, $K_{\max}^{i}=15,\;K_{\max}^{e}=10$ are selected to correspond to the minimum wavelengths of magnetic fields in the horizontal direction, which are approximately 200 and 250 km, respectively. The Mehler function mainly provides a stable space continuation in the radial direction to prevent the shock effect of the model in the data gap layer (340~450 km). After testing, $P_{\max}=\;5$ is set, and this can balance the data resolution in radials and the fitting in each height layer.

### 4.2. Spherical Cap Splicing

**Figure 5:**
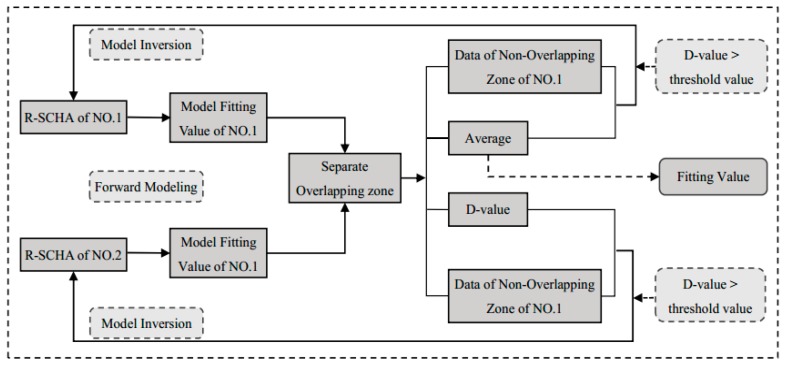
Flowchart showing the processing procedure for the overlapping area of the adjacent spherical caps.

An overlapping area of approximately 7.8° exists between adjacent spherical caps. In the overlapping area, certain differences exist in the geomagnetic components fitted by R-SCHA models in two subregions. These differences involve splicing problems between adjacent spherical caps. The present paper initially utilizes the R-SCHA model to calculate the fitting value in grid spots in overlapped areas and analyze the mutual difference. If the mutual difference is greater than the given threshold value (i.e., 0.5 nT), then the mean fitting value of each subregion is regarded as the observed value. In addition, iterative inversion is conducted to gradually obtain fitted geomagnetic components in overlapped area grid spots in two subregions that are approximately equal. Finally, its mean value is taken as the final fitting result. Figure 5 shows the splicing flow of the fitting result of adjacent spherical caps.

### 4.3. Error Analysis

Considering that the inversion model adopts data sets of different heights, the intensity of the lithospheric magnetic field varies significantly at different height layers. Therefore, errors are fitted at CHAMP and Swarm satellite altitudes separately to analyze the consistency between the R-SCHA model fitting value and the original observation data. The model inversion coefficients in Regions 1 and 2 are used to calculate the R-SCHA model inversion values in two regions and to seamlessly splice the overlapped region. The difference between the model inversion value and the original observation over the Tibetan Plateau and the root mean square error (RMSE) are calculated. Table 2 lists the statistical results of the errors of X, Y, and Z geomagnetic elements.

Table 2 shows that model fitting error decreases as altitude increases. The model fitting error in the CHAMP satellite altitude is slightly larger than that of Swarm satellite. However, the *RMSE* of all geomagnetic components are better than 0.3 nT.

**Figure 6:**
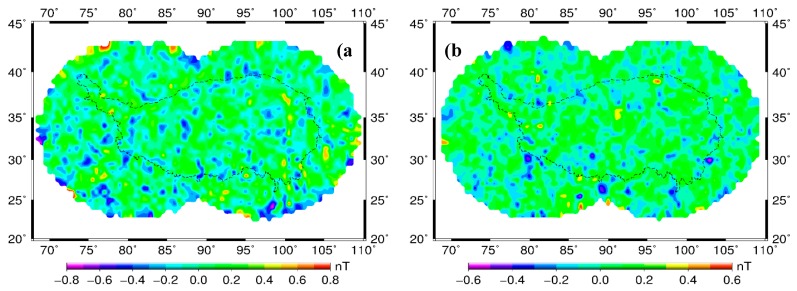
Contour maps of difference for Z component between R-SCHA model and satellite observation. (**a**) CHAMP satellite observation at 330 ± 5 km; (**b**) Swarm satellite observation at 470 ± 5 km.

Figure 6 shows the difference values (ΔZ) between the R-SCHA and CHAMP satellite at orbital altitude of 330 ± 5 km (a) and Swarm satellite at 470 ± 5 km (b). Figure 6 shows that the maximum errors are less than 0.8 and 0.6 nT at the height layers, respectively. This comparative analysis indicates that the inversion model is consistent with the original observation. The R-SCHA algorithm can accurately describe the distribution characteristics of the lithospheric magnetic field in the Tibetan Plateau. Figure 6 shows a slight shock in the boundary regions of two independent spherical caps. The shock may be related to the large extension degree of the Mehler basis function. On the edge of the spherical cap, the value of the Mehler basis function increases constantly with extension degree. This trend affects the fitting accuracy of the edge data. The lack of boundary data and uneven distribution also strongly influence the fitting effect.

**Figure 7:**
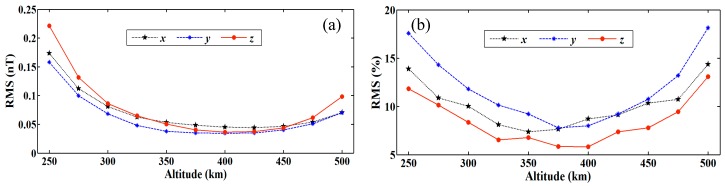
RMS distribution with altitude in nT (**a**) and in% (**b**) for X, Y, and Z components.

To further verify the stability of the model in the radial direction based on the model coefficient, the error between inversion model and EMM2015 (16°~720°) at an altitude range of 250~500 km every 25 km is computed based on a 0.5° × 0.5° grid resolution. Given that the absolute error of the model decreases as height increases, RMSE (in %) is used to show the statistical result in Figure 7b. Figure 7 does not indicate an obvious increase of errors (in %) from 350 km to 450 km, although the data lack constraints at these intermediate altitudes. The R-SCHA model can provide a stable spatial continuation in the radial direction with a reasonable setting of the truncation degree.

### 4.4. Geological Structures Analysis of Magnetic Anomalies in the Tibetan Plateau

**Figure 8:**
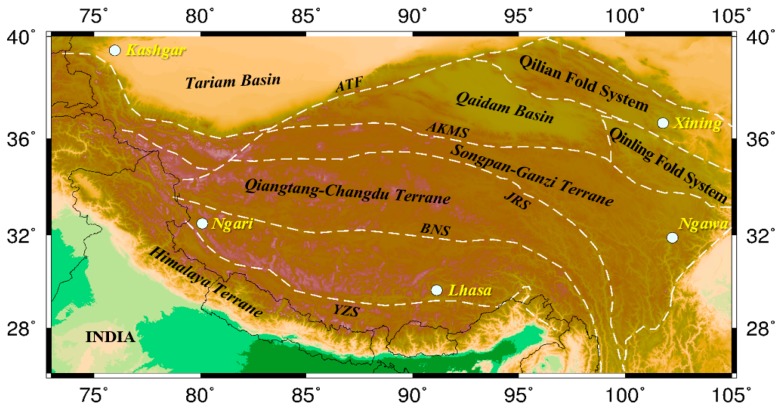
Tectonic map of the Tibetan Plateau. AKMS (Ayimaqin-Kunlun Mutztagh suture); ATF (Altyn Tagh fault); BNS (Bangong-Nujiang suture); JRS (Jinsha River suture); YZS (Yarlung-Zangbo suture).

The geological structures of the Tibetan Plateau are complex and have a unique and deep tectonic background. The boundary between the northern edge of plateau and the Tarim Basin is the Altun Tagh fault zone. The northeast part of the Tibetan Plateau is the Qaidam Basin. The plateau mainly includes the Songpan-Ganzi block, the Qiangtang–Chengdu block, the Lhasa block, and the Himalaya block from north to south. These blocks are bounded by the LungmuCo-Jinshajiang suture zone, the Bangong-Nujiang suture zone, and the Yarlung-Zangbo suture zone [52,53] (Figure 8).

**Figure 9:**
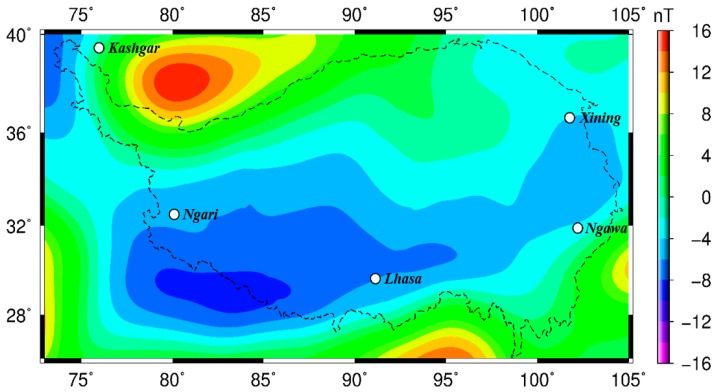
Contour map of Z component of the lithospheric magnetic field at 300 km altitude.

To analyze the relationship between the magnetic anomalies and geological structures of the Tibetan Plateau, a contour map of the lithospheric magnetic field in the Tibetan Plateau at a height of 300 km based on 0.5° × 0.5° grid resolution is first drawn (Figure 9). The red region in the figure represents the positive anomaly, and the blue region denotes the negative anomaly.

The large-background lithospheric magnetic field in the hinterland of the Tibetan Plateau shows the negative anomalies that formed a sharp contrast with positive anomalies observed in the Tarim Basin located in the north, the Sichuan Basin located in the east, and the Indian subcontinent located in the southwest. This demonstrates that an immense difference exists in the basement tectonics of the Plateau hinterland and the surrounding blocks. The midwestern part of the weak magnetic anomaly region is approximately distributed in a strip shape along the east–west direction. This distribution is basically consistent with the orientation of the regional geological structures, and its boundary basically agrees with the boundary of regional tectonic. However, in the east of the plateau, the magnetic anomaly gradually extends along the northeast direction, thereby exhibiting an arc shape. Thus, the magnetic anomaly is inconsistent with the east–south distribution of the geological structures zone. This phenomenon indicates that the anomaly in the east of the plateau may be caused by the magnetic materials in the middle and shallow layers of the earth’s crust.

The satellite magnetic anomaly map shows that the magnetic anomaly intensity and its distribution in all secondary structures zones in the Tibetan Plateau from the north to the south differ. A regional clump by positive anomaly is distributed in the middle part of the Tarim Basin. The western region adjacent to the Arkin fault zone shows a linear positive anomaly, but the eastern regions adjacent to the Qilian Mountain fault zone exhibit a negative anomaly. However, the positive and negative anomalies are weak. The Songpan-Ganzi block and the Qiangtang-Chengdu block are mainly manifested in a stable negative anomaly that approximates an east-west distribution. The eastern regions near the thrust nappe belt of the Longmen Mountains shows a northeast–southwest negative anomaly. Weak negative anomalies of these blocks imply that the Curie isothermal surface in these blocks is deeper than that in the south of the plateau. Magnetic anomalies in the Lhasa (Gangdise belt) and Himalaya blocks approximately show an east–west orientation along the west section of the Himalaya and across the Himalaya mountains, and this orientation continuously extends to the east region of Lhasa with a distinct negative anomaly. The strongest negative anomaly is located in the Himalaya region. The center of this anomaly is located approximately at 83.5° E and 29° N and has a center strength that reaches −9 nT. The negative anomaly may be a result of the strong collision between the blocks. Thrust nappes on the boundary of the block generate a friction phenomenon, thereby promoting constant increases in the temperature of deep crustal materials and the upward migration of hot materials. These effects lead to the Curie isothermal surface uplift. This explanation can be proved by the high heat flow activities in the Tibetan Plateau [54]. Therefore, a strong negative anomaly may imply that deep geological structures in this region are highly active.

## 5. Conclusions

Zonal revised of spherical cap harmonic analysis(R-SCHA) was used to model the lithospheric magnetic field of the Tibetan Plateau at satellite altitude. The accuracy and stability of this model were tested and evaluated in the horizontal and radial directions. Furthermore, the relationship between magnetic anomalies and geological structures was analyzed.

One of the key factors for modeling is refining satellite magnetic data. A complete data preprocessing procedure was designed, which involved data screening, model correction, track filtering, and hierarchical gridding. Reliability is another important factor of a model. Thus, the model must not generate significant shock effects in the horizontal and vertical directions but still achieve a certain spatial resolution. Considering that the difference between the latitude and longitude over the Tibetan Plateau is large, a small cap partition modeling can be used in a particular truncation level to provide good spatial resolution while ensuring accuracy. Therefore, the Tibetan Plateau was divided into two independent parts and the half-angle of the cap (approximately 1°) was moderately amplified to eliminate the influence of the boundary effect. Meanwhile, hierarchical gridding was conducted for satellite data distributed at a height of 250~510 km to guarantee the balance of each layer of data. The small semi angle of cap improved the fitting accuracy of the model throughout the region, but the difficulty of this method lies at the joint of each subregion model. The present paper used iterative inversion to create a method for seamless splicing between adjacent spherical caps. Moreover, model error and reliability analyses played important roles in the modeling process. The difference between the model value and the satellite observation value were initially calculated before the RMS of the model was quantitatively analyzed at the heights of the CHAMP and Swarm satellites. The fitting error of the model is small, and the RMS of each geomagnetic component is better than 0.3 nT. Moreover, the stability of the model in horizontal and vertical directions was examined by introducing the EMM2015 (16~720 order) high-accuracy lithospheric magnetic field model. The model does not have any significant shock effects in the horizontal direction. The model can provide a stable space continuation in the radial direction. The analysis indicates that the R-SCHA algorithm can accurately describe the middle- to long-scale lithospheric magnetic field in the Tibetan Plateau at the satellite altitude layer.

The separation of internal and external field sources remains a problem in the modeling process. The R-SCHA algorithm does not consider the joint estimation of the external source field. Instead, it facilitates the separation of an external field source through the model and filtering method in the data preprocessing steps. This method may introduce new errors in the process of model fitting. Therefore, Combining Swarm constellation differential gradient measurement is necessary to effectively suppress the interference of the long wavelength signal that remains in the external field and the main magnetic field. However, a map of a high-resolution lithospheric magnetic field over the Tibetan Plateau cannot be developed because of the lack of support for ground magnetic survey and aeromagnetic data. Although a good relationship between the distribution of magnetic anomalies and deep crustal tectonic structures at 300 km satellite altitude was identified, the model fails to extract abundant shallow geological structures information. Future research will be conducted on a downward continuation algorithm for geomagnetic anomaly data and multi-scale modeling will be integrated to obtain a high-resolution and high-accuracy lithospheric magnetic field. This endeavor will provide technical support for the study on the crustal structures of the Tibetan Plateau and related dynamic mechanisms.

## Figures and Tables

**Table 1:** Parameter values associated with R-SCHA inversion.

Region	Boundary		Pole of the Cone		Half Aperture of the Cone	Truncation Degree		
	Lower	Upper	Latitude	Longitude		$K_{\max}^{i}$	$P_{\max}$	$K_{\max}^{e}$
No. 1	Re+240km	Re+520km	33°	81°	10°	15	5	10
No. 2			33°	97°	10°	15	5	10

**Table 2:** Fitting errors of X, Y, and Z components on the height layer of CHAMP and Swarm.

Satellite Altitude	Item	ΔX/nT	ΔY/nT	ΔZ/nT
CHAMP (250~340km)	Min	–1.070	–1.419	–0.829
	Max	0.918	1.177	0.803
	RMS	**0.235**	**0.285**	**0.196**
Swarm (450~510km)	Min	–1.051	–0.882	–0.406
	Max	0.627	0.961	0.583
	RMS	**0.156**	**0.185**	**0.120**
